# Separation of conjoined spinal cords in symmetrical pygopagus twins using intraoperative neuromonitoring: pearls and pitfalls

**DOI:** 10.1007/s00381-023-05912-5

**Published:** 2023-03-23

**Authors:** Mohamed Ashraf Mahmoud, Sherif Bahaa Elshawady, George H. Korkar, Samar M. Hussein, Tarek H. Elserry, Mohamed Wael S. Mahmoud

**Affiliations:** 1 Neurosurgery department, Ain Shams University, Cairo, Egypt; 2 Physiology department, Suez Canal University, Ismailia, Egypt

**Keywords:** Pygopagus, Neuromonitoring, Conjoined twins, Separation

## Abstract

Pygopagus twin is a rare congenital malformation with a worldwide incidence of 1in 200,000. Few literature reports are published regarding the matter. In some cases, neuromonitoring is essential for safe surgical separation. We believe it is important to share our challenges and nuances in order to minimize obstacles one might encounter. We utilized neuromonitoring during our separation of both twins, and we planned a multidisciplinary approach and efficient communication system with the other teams in order to plan a successful, safe, and timely separation of the twins. We seek to highlight not our success but rather the obstacles and challenges we encountered during the separation of pygopagus twins in our institute using neuromonitoring for future reference.

## Introduction

The total reported worldwide prevalence of conjoined twins is 1.47 per 100,000 births approximately 50% of which are liveborn [1]. Pygopagus twin is a subtype of such congenital malformation that represents 6–19% of conjoined twins [2]. According to Hirokazu et al., there have been 31 sets of pygopagus twins described sufficiently in the past literature up to 2013, with noticed female preponderance (81%) [3].

Pygopagus twins may be fused at the skin, muscle, bone (sacrum or coccyx), dural sac, spinal cord, viscera, or reproductive organs. The conjoined spinal cord may be classified as V-shaped, Y-shaped, or U-shaped [4]. In the U-shaped spinal cord, it is more difficult to determine the anatomical midline. This often requires detailed investigations, special preoperative preparations, meticulous planning, and multidisciplinary participation from various specialties.

Since 1950, the number of successful separations of conjoined twins has been rising reported to be more than 200 [5]. The success of such separations relates largely to the shared anatomy of the two twins. There are only a few cases in the literature that reported cases of successful surgical separation of pyopagus twins that included spinal cords with the aid of neuromonitoring [4].

The author summoned this article to present the successful separation of a rare case of symmetrical pygopagus twins in our institute and highlight the challenges we encountered and had to overcome before, during, and after the procedure for future reference.

## Case presentation

**Fig. 1:**
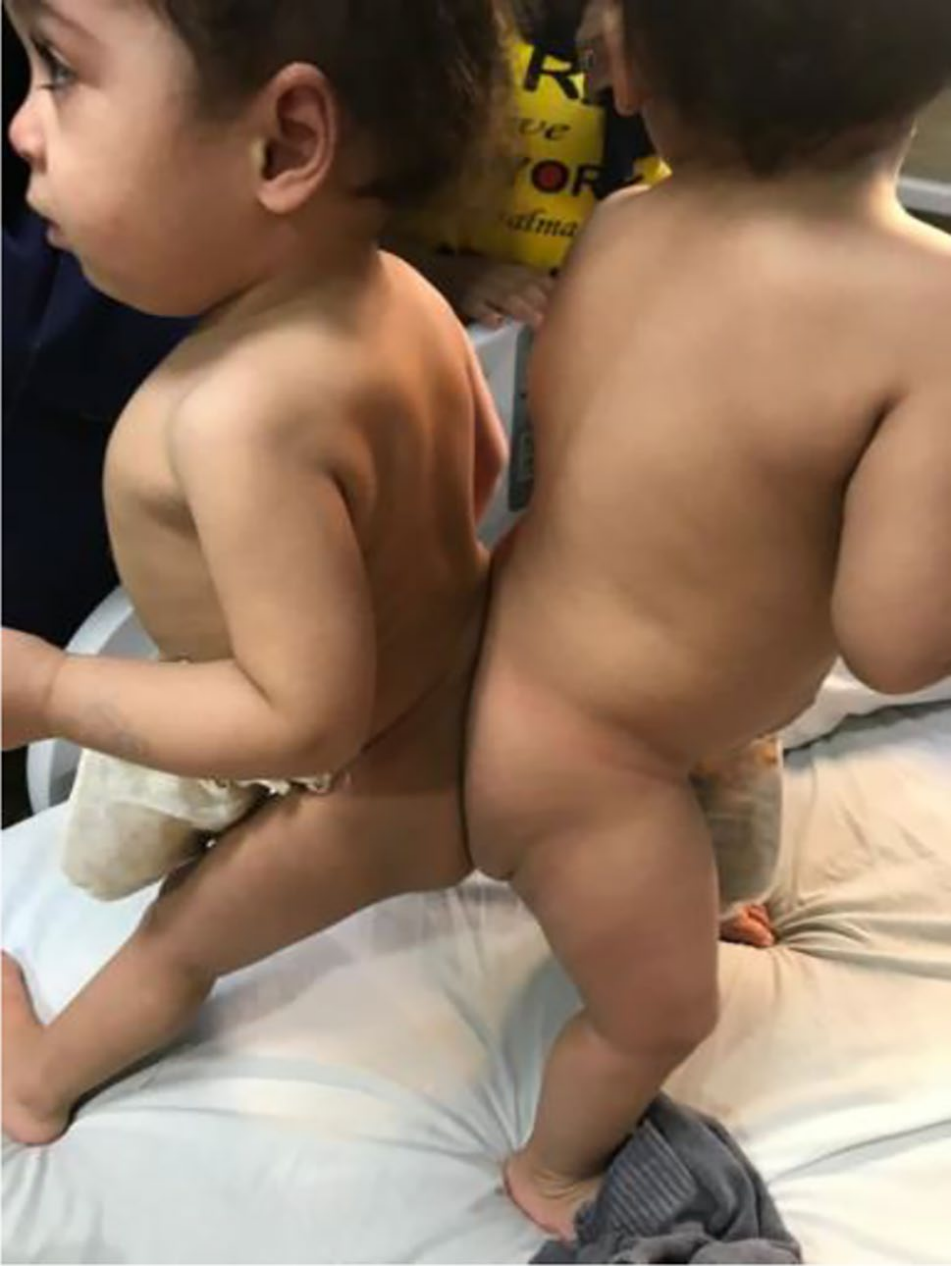
Picture of the two twins

Lean and Lana are 2 healthy young girls, aged 1 year and 7 months, respectively, shown in Fig. 1.

**Fig. 2:**
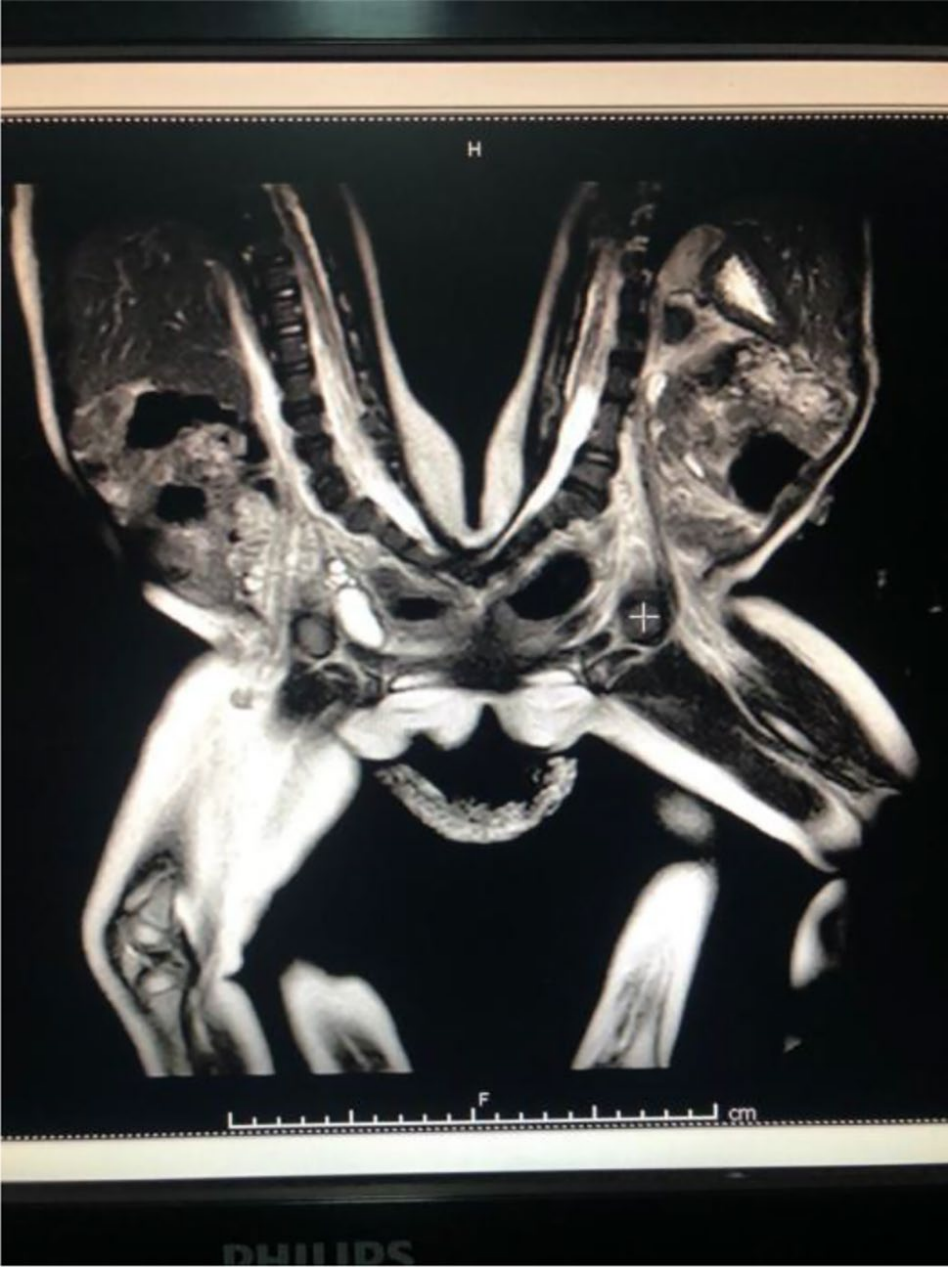
MRI of the pygopagus twins

They were prenatally diagnosed of their clinical condition as pygopagus twins during the third trimester, as MRI showed in Fig. 2.

They were delivered by cesarean section preterm and were managed in the NICU after delivery. At 2 weeks of age, one of the twins (Lean) developed gastrointestinal complications due to ano-vestibular fistulas and was arranged for a colostomy. Her clinical condition only partially improved after the colostomy but with frequent exacerbations. It was not until the other twin also underwent a colostomy procedure at the age of 8 months that subsequent improvement of both twins’ symptoms occurred.

Moreover, Lana showed a neurological motor deficit in her left lower limb for which MRI spine, electrophysiological nerve conduction, and electromyography were ordered. MRI results were consistent with the fused sacrum, the dural sac, and aberrant neural tissue.

Nerve conduction studies and electromyography studies were consistent with lumbosacral neuropathic weakness in the lower limbs with signs of reinnervation. Orthopedic length discrepancy of the lower limbs was diagnosed for which elective management after separation was decided.

We met the two twins at our institution in August 2020 for the planning of the separation procedure. Anesthesiologists, neurosurgery, neurosurgeons, plastic surgeons, pediatric surgeons, and orthopedic surgeons formed a team of multidisciplinary specialties who were responsible for decision-making and execution of the separation procedure.

## Preoperative planning

**Fig. 3:**
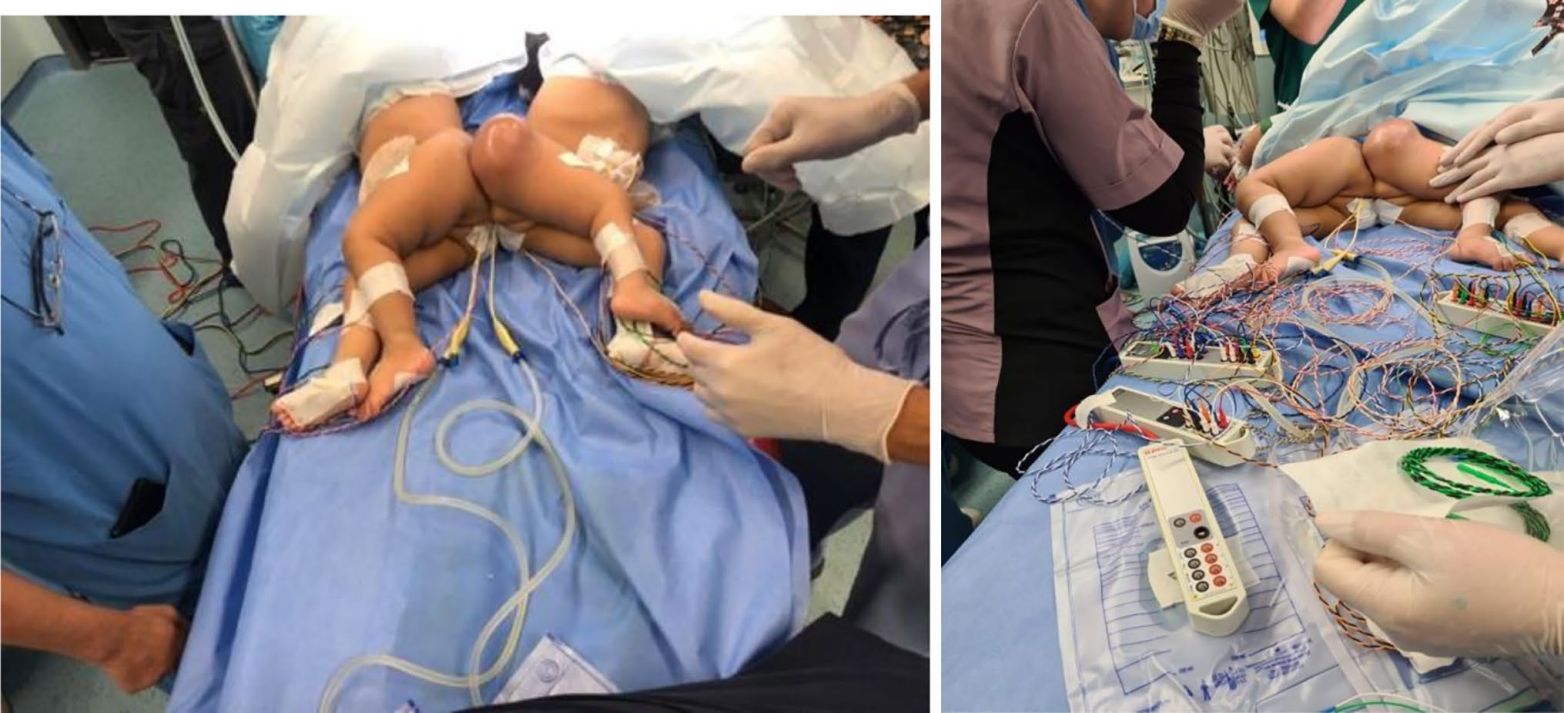
**a** Positioning of the twins on the operating room (OR) table; also shown is the tissue expansion device. **b** Intraoperative neuromonitoring device electrodes

A 4-month preoperative tissue expansion of the skin was recommended by plastic surgery before separation surgery. See Fig. 3.

The availability of 2 operating rooms and two teams from each specialty was prepared. A lengthy procedure was expected, and an early start was anticipated proper job delegation and description was assigned to each member for time efficiency.

The congestion of the operating room by personnel and adjuvant equipment (2 neurophysiological monitoring devices, 2 ventilators, and an operating microscope) was managed by team leaders to avoid traffic obstacles.

A large number of electrodes and cables for insertion and mounting were needed. Proper arrangement and efficient team communication were needed to avoid technical errors. Prompt handover of operating surgical teams was carried to maximize time efficiency and avoid complications. See Fig. 3b.

A strict infection control policy was applied to decrease of expected raised infection incidence due to the length of procedure, the number of personnel, the frequency handover of surgical teams, and transfer to different rooms after separation.

## Intraoperative procedure

**Fig. 4:**
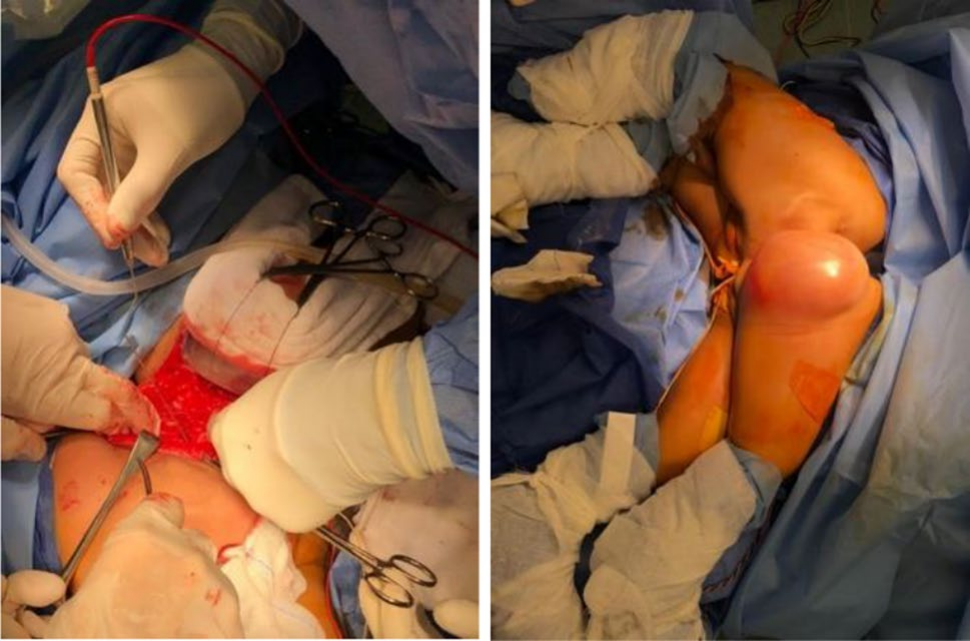
**a** Skin incision with the aid of stimulation for screening of neural structures

Plastic surgery planned the skin incision in order to achieve optimum skin reconstructive results as illustrated in the Fig. 4.

Our neurosurgical team continued with underlying tissue dissection guided by neuromonitoring. The lateral decubitus position after draping was a challenge for the anesthesia team during intubation. Furthermore, the lack of muscle relaxants as recommended by the neurosurgery team to avoid monitoring fallacies provided even more difficulties during intubation. The lateral decubitus also provided difficulties for the neurosurgical team as the anatomical orientation of the surgeon is usually in the prone position, ergonomics during dissection will be suboptimal, and finally, obscurity of the midline could lead to unnecessary complications.

Pediatric surgery was requested by the head of the neurosurgical team to attend and confirm the absence of visceral attachments or fistulous tracts in the dissection plane.

The technical data of the IONM used during dissection is mentioned below in detail with frequent stimulation to avoid severing aberrant neural tissue. After the fused sacrum, a bony bridge at the end of the vertebral column between the 2 twins was dissected and durotomy was done.

As the anatomical midline can be deceiving, the surgeons used neurophysiological stimulation along the axis of bridging tissue (bony, meningeal, and neural tissues) between twin A and twin B, and dissection was done along a plane where the surgeons did not elicit EMG responses from both twins.

Subsequent dissection by the pediatric surgery team and plastic surgery team was carried out for optimum preparation of anorectal and skin reconstruction, respectively.

Once the twins were separated, prompt transferal of one of the twins was carried out to the adjacent operating room where the second team of each specialty started to operate in the prone position. First dural reconstruction by neurosurgery, followed by anorectal reconstruction by pediatric surgeons, and finally skin reconstruction by plastic surgeons.

### Intraoperative neuromonitoring plan

The neuromonitoring plan comprised of two intraoperative neuromonitoring (IONM) devices, 8 channels of ISIS expert and Xpress systems (Inomed Medizintechnik GmbH, Emmendingen, Germany), one for each twin. We assigned one device for Lean (twin A) and the other device for Lana (twin B).

Multimodal scenarios were integrated into the protocol of the surgery monitored by our team of two neurophysiologists one regulating each device. The IONM protocol included free-run electromyography (fEMG), triggered EMG (tEMG), transcranial motor evoked potential (TcMEPs), and somatosensory evoked potentials (SSEPs).

The neurophysiology team has requested total intravenous anesthesia (TIVA) protocol for the ease recording of EMG and TcMEPs. A single dose of short-acting muscle relaxant was given to both of the twins for intubation at the induction phase of anesthesia. The maintenance protocol comprised of propofol and fentanyl, doses of which were modified according to the requirements and stage of surgery. Stable arterial blood and blood and body temperature were maintained throughout the surgery.

### Electromyography

**Fig. 5:**
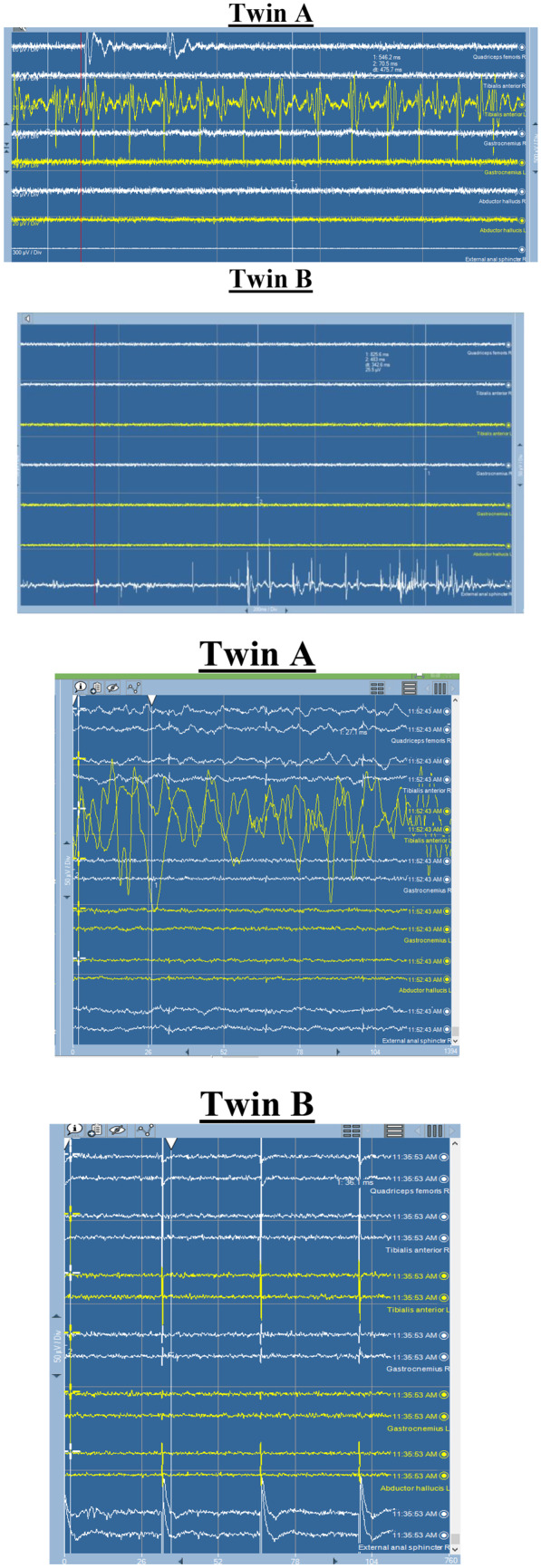
**a** EMG recordings from both twins. A, fEMG showed train activity of TA from twin A concomitant with EAS firing from twin B, the alarm which was reported to the surgeons. B shows tEMG responses recorded from direct stimulation of suspected neural tissue confirming responses of TA from twin A and EAS from twin B. **b** EMG recordings from both twins. A: fEMG showed train activity of TA from twin A concomitant with EAS firing from twin B, the alarm which was reported to the surgeons. B: shows tEMG responses recorded from direct stimulation of suspected neural tissue confirming responses of TA from twin A and EAS from twin B. fEMG, free running electromyography; tEMG, triggered electromyography; TA, tibilais anterior muscle; EAS, external anal sphincter.

EMG activity was monitored from the bilateral lower limb muscles of each twin to detect any significant simultaneous stimulation bridging neural tissue. fEMG and tEMG recording and stimulation parameters are summarized in Table 1. Data were recorded using twisted subdermal needle (SDN) electrodes, which had impedance check below 3 kΩ. Surgeons were informed about any alarming signs of neurotonic discharge which may indicate any compromise to the neural structure.fEMG train activities were recorded from the tibialis anterior (TA) muscle of twin A and the external anal sphincter (EAS) of twin B (Fig. 5A), during the stage of division of the neural structures. At this stage, the dissection was performed under the umbrella of sequential tEMG of all tissues before severing them.tEMG stimulation at 0.5 mA confirmed grouped responses of TA muscle in twin A and EAS in twin B (Fig. 5B), which suggested overlapping innervation between the twins.

**Table 1:** fEMG and tEMG parameters

fEMG Recording	— Bilateral quadriceps femoris, tibialis anterior (TA), gastrocnemius (GC), abductor halluces (AH) muscles and external anal sphincter (EAS).
	— Ground SDN electrode is placed at one shoulder
	— Sensitivity: 100 uV/div
	— Time base: 200 ms/div
tEMG stimulation	Concentric bipolar stimulation probe (Inomed, Emmendingen, Germany)
	— Parameters:
	— Intensity: 0.5–4 mA
	— Pulse type: single
	— Frequency: 3 Hz
	— Pulse width: 200 us
	— Sensitivity: 50 uV/div
	— Time base: 20 ms/div
Filter settings	LPF: 30 Hz
	HPF: 1000 Hz

*fEMG* free running electromyography, *tEMG* triggered electromyography, *SDN* subdermal needle electrodes, *LPF* low pass filter, *HPF* high pass filter

### Transcranial motor evoked potential

TcMEPs monitoring was performed to evaluate the integrity of the motor tracts of both twins. Recording and stimulation parameters are mentioned in Table 2. The alarming sign was the attenuation of TcMEPs amplitude of more than 50% from the baseline amplitude (8). Baseline TcMEPs were taken before skin incision and showed good responses for all muscles of both twins except for left TA, GC, and AH of twin B which showed no responses. Follow-up recordings were performed after nerve root separation. No alarming changes were noted in TcMEPs till the end of surgery.

**Table 2:** TcMEPs recording and stimulation parameters

Recording	Bilateral quadriceps femoris, tibialis anterior (TA), gastrocnemius (GC), abductor halluces (AH) muscles and external anal sphincter (EAS).
	Ground SDN electrode is placed at one shoulder
Stimulation	Corkscrew electrodes placed at C1-C2 according to the 10-20 international system of EEG recording
	— Intensity: 150 mA
	— Pulse type: train of 5 pulses
	— Frequency: 1 Hz
	— Pulse width: 500 us
	— ISI: 4 ms
	— Sensitivity: 50 uV/div
Filter settings	LPF: 200 Hz
	HPF: 2000 Hz

*SDN* subdermal needle electrodes, *EEG* electroencephalogram, *ISI* interstimulus interval, *LPF* low pass filter, *HPF* high pass filter

### Somatosensory evoked potentials

SSEPs recording and stimulation parameters are summarized in Table 3. Baseline amplitude and latency of cortical SSEPs were recorded before the skin incision and follow-up SSEPs were recorded every 5–10 min. The alarming criteria of SSEP are amplitude decrease of 50% and/or latency increase more than 10% compared to baseline (8). No alarming SSEPs changes were noted throughout the surgery. The authors were aware of limitations of SSEPs when dealing with pure motor nerve fibers and routinely run MEP check-ups before critical dissection steps.

**Table 3:** SSEPs recording and stimulation parameters

Stimulation	— Upper SSEPs: Median nerve
	— Lower SSEPs: Posterior tibial nerve
	— Ground SDN electrode at one of
	the shoulders
	— Stimulation intensity:
	10 mA for upper SSEPs and 20 mA for lower SSEPs
	— Pulse width: 200 usec
	— Repetition rate:
	4.7 Hz for upper SSEPs and 3.7 Hz for lower SSEPs
	— Averaging: 200-300
Recording	— Upper SSEP-s: C3'-Fpz and C4'-Fpz
	— Lower SSEPs: Cz'-Fpz
	according to the 10-20 international system of EEG recording
Filter settings	LPF: 30 Hz
	HPF: 500 Hz

*SSEP* somatosensory evoked potentials, *SDN* subdermal needle electrodes, *EEG* electroencephalogram, *LPF* low pass filter, *HPF* high pass filter

## Postoperative results

**Fig. 6:**
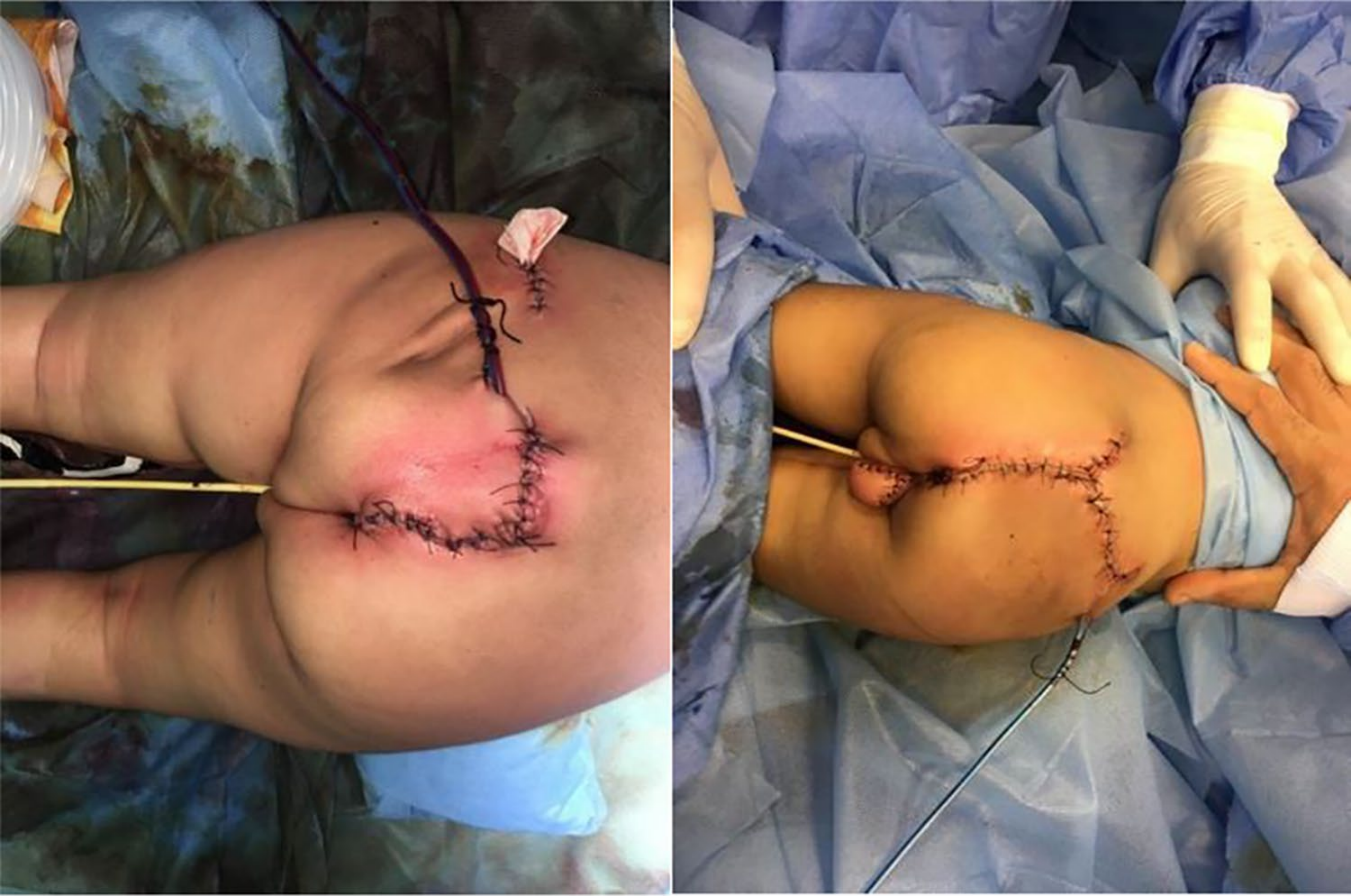
Postoperative wounds, drains, and catheters of the twins

Wound closure was performed by the plastic surgery team as shown in Fig. 6. Postoperative recovery of the two twins was uneventful, with no delayed recovery from anesthesia, no postoperative blood transfusion was needed, drains showed no signs of CSF leakage, wounds healed uneventfully, no added neurological deficit of the lower limbs was noticed on both twins, a few months later colostomies were closed with normal bowel habitus of both twins, and finally one of the twins is undergoing the orthopedic procedure for lower limb length discrepancy.

## Discussion

There is a paucity of reports in the literature that discusses pygopagus twins with the fused sacrum and U-shaped spinal cord. Hockley et al. [6] reported a similar case in 2004 which was successfully managed, however, at much earlier age than our case. Anesthesia considerations were highlighted. Angiography for cross-circulation was considered but was not done as the surgical separation was urgent. In our case, the twins were older, and such an issue was not addressed as adequate preoperative blood was prepared. Hockley et al. also reported a U-shaped malformation, as in our presented case, as opposed to a V-shaped malformation. It is more difficult to distinguish the anatomical midline in a U-shaped malformation as opposed to a V-shaped or Y-shaped malformation. Thus, this highlights the need to establish a functional midline during the neural separation of the two twins, in order to preserve neurological function and indicates neurophysiological monitoring.

For IONM, there was great attention when reporting any changes to the surgeons that the change happened in which twin. The major challenges for the IONM were the limited surface area for electrode placement and unclear anatomy of the external anal sphincters of both twins. IONM provides the ability to monitor for and possibly mitigate postoperative neurologic deficits. It also allows for the identification of neural elements with more definition than that provided by physical examination and imaging (7). IONM has served its goal in achieving safe dissection with minimal risk for postoperative neurological injury. This was done by combining tEMG and TcMEPs.

## Conclusion

Pygopagus twin is an uncommon form of the rare conjoined twin malformation. A few articles in the literature have discussed challenges faced when managing pygopagus twins. This article is aimed at highlighting challenges that the author has faced and subsequent management for future reference when faced with this entity. We believe careful multidisciplinary planning, communication, and decision-making, along with necessary adjuvant tools such as intraoperative neuromonitoring, are needed for the successful separation of such pathology without added morbidity. In addition, counseling of the mother for a lengthy preoperative course and lengthy procedure and meticulous postoperative follow-up is very important in order to meet the mother’s expectations. We hope that this case report contributes to the knowledge about the pygopagus twin’s separation.

## References

[1] Mutchinick OM, Luna-Muñoz L, Amar E, Bakker MK, Clementi M, Cocchi G (2011). Conjoined twins: a worldwide collaborative epidemiological study of the International Clearinghouse for Birth Defects Surveillance and Research. Am J Med Genet Part C Semin Med Genet.

[2] Spitz L (2005). Conjoined twins. Prenat Diagn.

[3] Hirokazu T, Takayuki I, Yoshinori H, Kazunari K, Akio A, Keiji K (2013). Separation surgery of pygopagus asymmetrical conjoined twins sharing U-shaped spinal cord: case report and literature review. Child’s Nerv Syst.

[4] Yokota C, Kagawa N, Bamba Y, Tazuke Y, Kitabatake Y, Nakagawa T (2021). Successful neurosurgical separation of conjoined spinal cords in pygopagus twins: illustrative cases. J Neurosurg Case Lessons.

[5] Frawley G (2020). Conjoined twins in 2020 - state of the art and future directions. Curr Opin Anaesthesiol.

[6] Hockley AD, Gornall P, Walsh R, Nishikawa H, Lam H, MacPherson L (2004). Management of pyopagus conjoined twins. Child’s Nerv Syst.

[7] Cromeens BP, McKinney JL, Leonard JR, Governale LS, Brown JL, Henry CM (2017). Pygopagus conjoined twins: a neurophysiologic intraoperative monitoring schema. J Clin Neurophysiol.

